# What do women with gynecologic cancer know about HPV and their individual disease? A pilot study

**DOI:** 10.1186/1471-2407-14-388

**Published:** 2014-05-30

**Authors:** Sophie Pils, Elmar A Joura, Max-Paul Winter, Anup Shrestha, Agnes Jaeger-Lansky, Johannes Ott

**Affiliations:** 1 Department of Obstetrics and Gynecology, Medical University of Vienna, Waehringer Guertel 18-20, 1090 Vienna, Austria

**Keywords:** HPV, Awareness, Disease knowledge, Patient information, Cancer

## Abstract

**Background:**

The vaccinations against human papilloma virus (HPV) are highly effective in preventing persistent infection. The level of knowledge about HPV and the consequences of an infection with this virus are low in the general population and in patients who suffer from HPV-associated diseases. We aimed to compare the level of knowledge about HPV and about the women’s individual malignant disease between women with and without HPV-associated gynecologic cancer as well as the knowledge about individual malignant diseases.

**Methods:**

In a pilot study, 51 women with HPV-related cancer (cervical cancer: n = 30; vulvar or vaginal cancer: n = 21) and 60 women with non-HPV associated gynecologic malignancies (ovarian cancer: n = 30; endometrial cancer, n = 30) were included. They answered a questionnaire including questions about personal medical history, risk factors for cancer development, and HPV.

**Results:**

The general level of knowledge of the term “HPV” was low (29.7%, 33/111) and it was similar in patients with HPV-related and non-HPV-associated cancer (18/60, 30.0% vs. 15/51, 29.4%, respectively; p = 1.000). When asked about their disease, 80% (24/30) of women with ovarian cancer correctly named their diagnosis, followed by women with cervical cancer (73.3%, 22/30), endometrial cancer (70%, 21/30) and vaginal or vulvar cancer (42.9%, 9/21; p = 0.008).

**Conclusion:**

The level of knowledge about HPV and the malignant diseases the patient suffered from was low. This applied even to patients with HPV associated malignancies.

## Background

Malignancies of the female urogenital tract are among the most common causes of death in women. In addition to adequate medical and surgical treatment, women need detailed information about the risk factors, therapeutic options, including alternatives to standard treatments, as well as possible complications and side effects [1,2].

Several risk factors for cancer have now been identified. These include age, body mass index (BMI), viral infections, and genetic factors, such as BRCA1 and BRCA2 mutations [3-6]. Both the discovery of the human papilloma virus (HPV) as the main trigger for cervical cancer, and the development of an immunization against these viruses, were groundbreaking in the field of gynecologic oncology. HPV has also been identified as a risk factor for various other malignant diseases, such as vulvar cancer, anal cancer, and oral cancer [7-9].

The vaccinations against human papilloma virus (HPV) are highly effective in preventing persistent infection and are well tolerated by patients [10]. The HPV immunization enables the active prevention of a malignant disease for the first time. Nonetheless, the level of knowledge about HPV and the consequences of an infection with this virus are low in the general population [11,12]. To date there is no study on knowledge concerning HPV in patients who suffer from HPV-associated malignancies.

If a woman is aware of the details of her disease, including information about risk factors, therapeutic options, and consequences, she might find it easier to make her own decisions concerning further treatment options.

Moreover, this knowledge might help in coping with the disease. Thus, we consider it a necessity to transmit our knowledge to these patients and make it available to them. In other diseases, it has been shown that an increase in disease-specific knowledge augments medical and surgical treatments by improving quality of life [13,14]. Although this observation has not been reported in gynecological malignancies as yet, it seems that this would be true for this patient cohort as well. In addition, well-educated patients may have a positive impact on disease awareness in the general population, because they may share their knowledge concerning HPV with friends and relatives and, in doing so, help to enhance effective screening. Additionally it may be of interest to reach the male population in order to maintain consistent information of all patients.

The main study objective was to compare the level of knowledge about HPV between women with HPV-associated cancer and women with other gynecologic malignancies. As a second study objective, we aimed to evaluate the level of knowledge about general risk factors that promote cancer development, as well as the level of knowledge about individual malignant diseases.

## Methods

### Study design and patient population

The study was based on the hypothesis that the vast majority of patients who suffer from HPV-associated cancer do not know of the association between HPV and the development of urogenital malignancies. In this pilot study, 51 women with HPV- or possibly HPV-related cancer (cervical cancer: n = 30; vulvar or vaginal cancer: n = 21) and 60 women with non-HPV associated gynecologic malignancies (ovarian cancer: n = 30; endometrial cancer, n = 30) were included from January 2012 to May 2012. All of these patients were treated at the Department of Obstetrics and Gynecology of the Medical University of Vienna. Only patients who were fluent in written and spoken German were included. To ensure a fast and clean data collection in this pilot study, we aimed to enroll 30 patients per disease type. Due to the low number of patients with vulvar or vaginal cancer, we were unable to enroll 30 women with these types of cancer (n = 21). The ethics committee of the Medical University of Vienna approved the study (IRB number 1202/2011).

Patients were asked to participate in the study during the course of their regular follow-up visits at the hospital-based outpatient clinic for gynecologic oncology. After they gave written, informed consent, they had to answer a questionnaire that included questions about personal medical history, risk factors for cancer development, and HPV (see Additional file 1). The questionnaire had been developed by the study team for this specific study and, thus, had not a validated before. The patients answered the questions in the presence of a study physician that helped the patients in cases of ambiguities. Due to this close contact, we were able to obtain fully answered questionnaires.

The scope of the questionnaire design was to keep the questions simple, to provide a reliable overview on the general knowledge of the diseases in our patients. The questionnaire was then approved by the ethics committee of the Medical University of Vienna.

For the “overall treatment” and “risk factors” questions, answers were scored as “correct” when all treatment options had been identified correctly. Answers were scored as “partially correct” if there was one mistake (one treatment option had been forgotten or too many had been specified), and scored as “wrong” if there were two or more mistakes. For removed structures or organs, answers were scored as “correct” if all removed structures had been specified correctly, “partially correct” if there were one or two mistakes, and “wrong” if there were three or more mistakes. The patients had to correctly identify the following risk factors for their individual disease: smoking and infection with HPV for cervical cancer; smoking, infection with HPV, diseases of the skin like lichen sclerosus or vulvar intraepithelial neoplasia for vaginal/vulvar cancer; a family predisposition and a high body mass index for ovarian cancer; and a high body mass index and diabetes for endometrial cancer.

Data about patients’ oncologic treatment were reviewed by retrospective chart analysis.

### Statistical analysis

Variables are described by frequencies and mean ± standard deviation (SD). Differences in categorical parameters were tested using contingency tables. Metric parameters were analyzed using the Kruskal-Wallis-test. Correlations were tested using Spearman’s rank test. A p-value <0.05 was considered significant. Statistical analysis was performed using SPSS 15.0.1 for Windows (SPSS Inc., 1989–2006).

## Results

Basic patient characteristics are provided in Table 1. The general recognition of the term HPV was low (29.7%, 33/111). There was no statistical difference in the time between the diagnosis and the interview between the different disease entities. The median time between diagnosis and inclusion into our study was 3.9 years.

**Table 1:** Basic patient characteristics in women with HPV-associated and non-HPV-associated malignancies

		**Cervical cancer (n = 30)**	**Vaginal & vulvar cancer (n = 21)**	**Ovarian cancer (n = 30)**	**Endometrial cancer (n = 30)**	**P**
Age at the survey (years)*	51.87 ± 9.97	63.52 ± 14.46	60.70 ± 10.06	68.37 ± 9.34	0.001
Years since diagnosis*	3.55 ± 3.19	4.73 ± 3.99	4.58 ± 4.07	2.95 ± 2.2	0.361
Pre-therapeutic BMI (kg/m^2^)*	29.20 ± 9.06	28.984 ± 8.41	27.49 ± 4.95	28.48 ± 5.89	0.576
Post-therapeutic BMI (kg/m^2^)*	27.54 ± 7.29	28.12 ± 6.59	27.16 ± 5.74	28.60 ± 5.49	0.577
Correctly named diagnosis^#^	22 (73,3)	9 (42,9)	24 (80)	21 (70)	0.008
Educational level					
No school graduation^#^	1 (3.3)	1 (4.8)	0	1 (3.3)	0.86
Secondary school graduation^#^	18 (60)	16 (76.2)	18 (60)	21 (70)	
High school graduation^#^	4 (13.3)	2 (9.5)	3 (10)	4 (13.3)	
Academic degree^#^	7 (23.3)	2 (9.5)	9 (30)	4 (13.3)	

Values are provided as *mean ± standard deviation or ^#^number (per cent). Parameters were tested using *Kruskal Wallis tests or ^#^contingency tables.

When comparing the knowledge of HPV with the knowledge of the type of disease/the removed structures/the overall treatment (0 = incorrect, 1 = partially correct, 2 = completely correct), no correlations were found (p = 0.070, p = 0.108, and p = 0.051; respectively). Moreover, the knowledge of HPV did not differ between the cancer types (ovarian cancer 9/30, 30%; cervical cancer, 11/30, 36.7%; endometrial cancer 7/30, 23.3%; with vaginal or vulvar cancer 6/21, 28.6%).

When asked about their disease, 80% (24/30) of women with ovarian cancer correctly named their diagnosis, followed by women with cervical cancer (73.3%, 22/30), and endometrial cancer (70%, 21/30). Only 42.9% of patients with vaginal or vulvar cancer could identify their illness (9/21; p = 0.008) (Table 1). When focusing on knowledge about structures that had been removed during surgery, women who suffered from ovarian cancer revealed the lowest level of knowledge (20% = 6/30 completely correct answers, 56.7% = 17/30 partially correct answers, 23.3% = 7/30 incorrect answers; Table 2).

**Table 2:** Level of knowledge about cancer-related treatment according to the type of carcinoma

	**Correct**	**Partially correct**	**Incorrect**	**p**
Overall treatment				
CC	80.0	6.7	13.3	0.066
V&VC	81.0	9.5	9.5	
	OC	93.3	6.7		0.0
	EC	86.7	13.3		0.0
Operation (removed structures)					
CC	63.3	33.3	3.3	<0.001	
V&VC	47.6	28.6	23.8		
OC	83.3	0.0	16.7		
EC	63.3	30.0	6.7		
Risk factors					
CC	0.0	26.7	72.4	0.925	
V&VC	0.0	23.8	76.2		
OC	0.0	16.7	83.3		
EC	0.0	46.7	53.3		

CC = cervical cancer; V&VC = vaginal & vulvar cancer; OC = ovarian cancer; EC = endometrial cancer.

Data are provided as frequencies and percentages. Differences were tested using contingency tables.

We then compared women with and without HPV-related cancer. The latter were significantly older (64.5 ± 10.4 vs. 56.7 ± 13.2 years, p < 0.001). The level of knowledge of the term “HPV” was the same in patients with HPV-related cancer (18/51, 35,3%) as in patients with non-HPV-associated neoplasia (15/60, 25%, p = 0,097). Patients with HPV-associated cancer did not know their exact diagnosis significantly more often (p = 0.044; (Table 3). There were no significant differences in the level of knowledge about cancer-related treatment and risk factors (Table 2).

**Table 3:** Knowledge on cancer in women with HPV-associated and non-HPV-associated malignancies

	**HPV-related cancer (n = 51)**	**HPV non-related cancer (n = 60)**	**P**
Age at the survey (years)*	64.5 ± 10.4	56.7 ± 13.2	0.001
HPV recognition^#^	18 (35,3)	15 (25)	0.097
Knows diagnosis^#^	30 (58,8)	46 (76,7)	0.044

Values are provided as *mean ± standard deviation or ^#^number (per cent). Parameters were tested using *Kruskal Wallis tests or ^#^contingency tables.

We observed a significant correlation of the educational level (ranging from 0 to 3) and knowledge concerning HPV (Spearman’s rho, correlation coefficient 0.421, p < 0.001). There was no significant difference in educational levels between patients with different cancer types (p = 0.763). Answers to questionnaire modules on risk and preventive factors according to the type of disease are provided in Additional file 2: Table S1.

The vast majority of patients reported that doctors had spent enough time with them to answer all their questions. There were no differences in satisfaction about time spent on the individual patient between women with cervical cancer (30/30, 100%), vaginal/vulvar cancer (2/21, 9.52%), ovarian cancer (2/30, 93.3%), and endometrial cancer (2/30, 93.3%) (p = 0.962). There was no difference in knowledge concerning HPV between patients who were satisfied with the time spent by our physicians on their cases and those who were not (p = 0.144).

## Discussion

The diagnosis of a malignant disease is a dramatic life event for most affected people. Despite the fact that malignant diseases affect patients’ quality of life in a substantially negative fashion, women who suffer from malignant diseases of the female genital tract reveal quite a low level of knowledge about their disease and its development.

One of the milestones in gynecologic oncology was the identification of HPV as a co-factor in the development of malignant diseases [15]. This finding led to the development of a vaccination against HPV in order to prevent associated malignancies [16-18]. Although this was unparalleled, many people are still unaware of HPV and the impact of an infection. Donders et al. demonstrated that only about 50% of women who attended a routine gynecologic hospital-based outpatient clinic were aware of the role of HPV in cervical cancer and the possibility of getting a vaccination to prevent it [11]. This was in accordance with a Dutch study that included nearly 700 female and male students who were interviewed about HPV, cervical carcinoma, and HPV vaccine acceptance. Notably, the study was performed after implementation of the HPV vaccine in the Dutch national vaccination program. More than 50% of the students lacked knowledge about HPV [19]. In our study population, the level of knowledge was similar. Depending on the type of cancer, 23.3% to 33.3% of patients knew the term HPV”, and there was an association between a low level of education and lack of knowledge about HPV.

Nevertheless, the general level of knowledge about HPV seems to have increased in the past several years. This was suggested by the results of a systematic review of parental surveys about HPV and/or child HPV-vaccination. The percentage of parents who had heard about HPV rose over time (from 60% in 2005 to 93% in 2009), as did their appreciation for the HPV infection and cervical cancer link (from 70% in 2003 to 91% in 2011) [20]. Whether this rise in the level of knowledge will also occur in women with these types of cancer, as in our study population, is the focus of further studies in our department. In contrast to previous studies we aimed to investigate the knowledge of cancer patients and not of the general population. Furthermore, we chose a timespan (January 2012–May 2012) in which the vaccination had already been implemented (Oct. 2006) and well-established in Austria. To the best of our knowledge, this has not been investigated in comparable surveys.

We carefully evaluated whether our patients were satisfied with the time spent by our physicians on their cases to provide medical information and answer questions. Since this was the case, the low level of knowledge was considerably not attributable to a lack of time spent with patients. Moreover, patient satisfaction and knowledge were not associated with each other. Independent of the underlying malignancy, the vast majority of study participants was satisfied with the amount of time that was spent on their individual care. Nonetheless, our data demonstrate that women with gynecologic malignancies have a low overall knowledge of their disease and of their individual treatment options. Notably, when focusing on whether patients knew their diagnosis, women with vaginal or vulvar cancer were able to give the correct answer less than 50% of the time, compared to 73–80% in the other groups. Our survey revealed similar data when patients were asked about structures that had been removed during surgery. Again, only 47% of patients with vaginal or vulvar cancer knew these details, compared to at least 63% of patients with other malignancies. It is possible that, in general, patients might not be aware of the difference between vagina and vulva, which could explain these results in our study population. This assumption about a lack of anatomical knowledge is supported by the fact that there was no difference between the groups concerning the level of knowledge about the treatment modalities they had received (chemotherapy, irradiation, immunotherapy, hormone therapy, surgery). Rather alarmingly, only 33.3% of our patients with an HPV-associated cancer knew exactly what HPV was. This is a much lower rate than reported by Donder or other authors [11,12,21]. We think that this lack of knowledge in our patients does not reflect patient education. Patients may feel stigmatized for having a disease due to a sexually transmitted infection and suppress the fact that their cancer may have been caused by this disease. Or they may have difficulties in dealing with the knowledge that their disease may have been preventable with lifestyle changes and vaccination.

Thus, our data suggest that patients need even more detailed information about the types of gynaecological cancers and the risk factors and causes of these diseases. The source of information for the patients is an important question. On the one hand it is the duty of national health care providers to supply the patients with information on preventable diseases. On the other hand we must not forget that the face-to-face setting of a patient-physician conversation may be the most effective way to keep patients informed. Nevertheless, recent research suggests that the information itself is important, not the source of information [21].

Moreover, the level of knowledge about risk factors associated with their cancer type was very low. None of the patients was aware of all known risk factors (Table 2). Women with cervical and ovarian cancer knew at least one risk factor (43.3% and 46.7%, respectively), and, thus, were more informed than women with vaginal/vulvar cancer (23.8%) or endometrial cancer (16.7%). We believe that patients need to be informed about their individual risk factors in more detail. One might assume that patients with malignancies – when well-provided with information about their disease – could contribute to the general medical education, since they possibly increase their relatives’ and friends’ awareness of risk factors.

We consider the fact that we were not able to use a standardized questionnaire about HPV [22] as a limitation. This is due to the fact that the vast majority of questionnaires were either too complex or were published after we had collected the data used in this report. Moreover, one might consider the small sample size problematic as well as the fact that only patients who were fluent in written and spoken German were included. However, the overall knowledge might be even lower in patients who are not native German speakers. Thus, we think that the major study conclusion is not limited by the latter draw-back. Another limitation of the study is its pilot nature. The authors wanted to gain a rough overview over the patients knowledge on HPV. The collected data will be used in following studies, in which a more extensive set of questions will be used to assess the knowledge of our patients precisely.

## Conclusion

In conclusion, cervical cancer is the second most common cancer in women and the leading cause of cancer-related death in many developing countries [1]. Screening for the precursors of this disease leads to a significant decline in mortality from cervical cancer in developed countries [23-25]. Despite these incredible advantages, we found that knowledge levels about HPV and cervical cancer were low, even in affected women. We believe that an educational campaign, supported by general practitioners, is needed to disseminate knowledge about HPV and cervical carcinoma [26]. To the best of our knowledge, the important barriers to vaccination are costs and a lack of information about the benefits of vaccination. It is likely that the removal of these barriers may spread immunization and result in a substantial decrease in HPV-related cervical disease, including cervical cancer.

## Competing interests

All authors declare that they have no political, personal, religious, ideological, academic, and/or intellectual competing interest.

Prof. Elmar Joura declares the following conflicts of interest: (i) Research funding by MERCK and GSK; (ii) lecture fees by MERCK, Sanofi Pasteur MSD, GSK, Roche; (iii) Advisory Board fees by MERCK. All other authors declare that they have no commercial interest, financial interest, and/or another relationship with manufacturers of pharmaceuticals, laboratory supplies, and/or medical devices or with commercial providers of medically related services.

## Authors’ contributions

Study design: All authors. Acquisition of data: SP, AS, AJ. Statistical analysis: MPW, JO. Writing of the manuscript: SP, EJ, AS, JO. Manuscript editing and final approval of the version to be published: All authors.

## Pre-publication history

The pre-publication history for this paper can be accessed here:

http://www.biomedcentral.com/1471-2407/14/388/prepub

## Supplementary Material

Additional file 1 Questionnaire-subsection. Click here for file

Additional file 2: Table S2 Answers to questionnaire modules on risk and preventive factors according to the type of disease. Click here for file
