# Supplementary material for: What do women with gynecologic cancer know about HPV and their individual disease? A pilot study
Source: BMC Cancer. 2014 May 30;14:388. doi: 10.1186/1471-2407-14-388 (PMC4046847; doi:10.1186/1471-2407-14-388)
Supplement: Additional file 1 — Questionnaire-subsection. [file 1471-2407-14-388-S1.docx]

Questionnaire-subsection

1. Before I got sick with cancer, I visited my gynecologist periodically:

• Once a year • Once every few years • Never

During these visits, the following examinations were conducted:

• PAP smear test • Ultrasound examination • Breast palpation and examination

2. I was diagnosed with the following disease:

• Ovarian cancer • Uterine cancer • Cervical cancer • Vulvar cancer • I can’t answer that

3. Which therapy was used to treat your disease (multiple answers possible):

• Chemotherapy • Irradiation • Immunotherapy (antibodies) • Hormone therapy

• Surgery • • I can’t answer that

4. Which organs were removed during the surgery?

• Uterus • Ovaries • Lips of the vulva •Uterine cervix •Lymph nodes •Intestine

• I wasn’t operated on • I can’t answer that

5. Based on your opinion, which of the following factors are associated with your disease?

• Smoking • Alcohol • Nutrition, overweight • Bacterial infections

• Genetic predisposition (family history) • Viral infections •Skin diseases •None of the mentioned factors

6. Do you know what human papilloma viruses (HPV) are?

Yes • No • I don’t know for sure but I have heard about it

7. Based on your opinion, which of the following factors influence HPV infections:

• Smoking • Overweight • Number of different sexual partners • Number of childbirths

• Vaccinations • I can’t answer that

8. What do you think you can do to prevent cancer:

• Nutrition • Vaccination • Sports • I can’t answer that

9. During my therapy, my doctors spent enough time with me to answer all my questions

• Yes • No

10. During my follow-up investigations, my doctors spent enough time to answer all my questions

• Yes • No

*Personal information:*

• How old are you?

• What is your highest level of education?

• What is your nationality?

• Did you smoke when you were diagnosed with cancer?

If yes, how many cigarettes per day?

• Do you smoke?

If yes, how many cigarettes per day?

• How tall are you?

• How much did you weigh before your disease?

• How much do you weigh now?

• Are there any cases of cancer in your family?

If yes, which of your relatives and what type of cancer?
