# Supplementary material for: What do women with gynecologic cancer know about HPV and their individual disease? A pilot study
Source: BMC Cancer. 2014 May 30;14:388. doi: 10.1186/1471-2407-14-388 (PMC4046847; doi:10.1186/1471-2407-14-388)
Supplement: Additional file 2: Table S2 — Answers to questionnaire modules on risk and preventive factors according to the type of disease. [file 1471-2407-14-388-S2.doc]

**Table S1.** Answers to questionnaire modules on risk and preventive factors according to the type of disease.

|  | Cervical cancer  (n= 30) | Vaginal & vulvar cancer (n= 21) | Ovarian cancer  (n= 30) | Endometrial cancer  (n= 30) |
| --- | --- | --- | --- | --- |
| ***1. Based on your opinion which of the following factors influence HPV infections*** | | | | |
| Smoking | 4 (13.3) | 1 (4.8) | 2 (6.7) | 2 (6.7) |
| Overweight | 0 (0) | 0 (0) | 2 (6.7) | 0 (0) |
| Number of different sexual partners | 7 (23.3) | 5 (23.8) | 5 (16.7) | 6 (20.0) |
| Number of childbirths | 0 (0) | 1 (4.8) | 1 (3.3) | 1 (3.3) |
| Vaccinations | 0 (0) | 0 (0) | 1 (3.3) | 1 (3.3) |
| I can’t answer that | 21 (70.0) | 14 (66.7) | 20 (66.7) | 23 (76.7) |
| ***2. What do you think you can do to prevent cancer*** | | | | |
| Lifestyle | 19 (63,4) | 15 (71,5) | 19 (63.3) | 18 (60,0) |
| Vaccination | 7 (23.3) | 5 (23.8) | 3 (10.0) | 3 (10.0) |
| I can’t answer that | 13 (43.3) | 11 (50.0) | 18 (60.0) | 18 (60.0) |
| ***3. How often do you visit your gynecologist*** | | | | |
| Every year | 20 (66.7) | 16 (76.1) | 19 (63.3) | 23 (76.7) |
| Once every few years | 6 (20) | 4 (19) | 10 (33.3) | 3 (10) |
| Never | 5 (16.7) | 1 (4.8) | 2 (6.7) | 4 (13.3) |
| **4. Smoking habits** | | | | |
| Smoking pre cancer diagnosis | 14 (46.7) | 6 (28.6) | 3 (10) | 7 (23.3) |
| Smoking post cancer diagnosis | 12 (40) | 3 (14.3) | 2 (6.7) | 4 (13.3) |
| **5. Family history of malignant disease** | 13 (43.3) | 9 (42.8) | 13 (43.3) | 21 (70.0) |

Data are provided as numbers and frequencies. Multiple answers were possible in modules 1 and 2.
